# The “TIDE”-Algorithm for the Weaning of Patients With Cardiogenic Shock and Temporarily Mechanical Left Ventricular Support With Impella Devices. A Cardiovascular Physiology-Based Approach

**DOI:** 10.3389/fcvm.2021.563484

**Published:** 2021-02-19

**Authors:** Carsten Tschöpe, Frank Spillmann, Evgenij Potapov, Alessandro Faragli, Konstantinos Rapis, Vivian Nelki, Heiner Post, Gunther Schmidt, Alessio Alogna

**Affiliations:** ^1^Department of Cardiology, Charité–University Medicine Berlin, Campus Virchow Klinikum, Berlin, Germany; ^2^Center for Regenerative Therapies (BCRT), Berlin Institute of Health (BIH), Charité–University Medicine Berlin, Campus Virchow Clinic, Berlin, Germany; ^3^German Center for Cardiovascular Research (DZHK), Partner Site Berlin, Berlin, Germany; ^4^Department of Heart Surgery, Deutsches Herzzentrum Berlin (DHZB), Berlin, Germany; ^5^Department of Internal Medicine and Cardiology, Deutsches Herzzentrum Berlin (DHZB), Berlin, Germany; ^6^Department of Cardiology, Contilia Heart and Vessel Centre, St. Marien-Hospital Mülheim, Mülheim, Germany

**Keywords:** cardiogenic shock, mechanical circulatory support, Impella, weaning algorithm, pressure-volume, conductance catheter

## Abstract

**Objectives:** Mechanical circulatory support (MCS) is often required to stabilize therapy-refractory cardiogenic shock patients. Left ventricular (LV) unloading by mechanical ventricular support (MVS) via percutaneous devices, such as with Impella® axial pumps, alone or in combination with extracorporeal life support (ECLS, ECMELLA approach), has emerged as a potential clinical breakthrough in the field. While the weaning from MCS is essentially based on the evaluation of circulatory stability of patients, weaning from MVS holds a higher complexity, being dependent on bi-ventricular function and its adaption to load. As a result of this, weaning from MVS is mostly performed in the absence of established algorithms. MVS via Impella is applied in several cardiogenic shock etiologies, such as acute myocardial infarction (support over days) or acute fulminant myocarditis (prolonged support over weeks, PROPELLA). The time point of weaning from Impella in these cohorts of patients remains unclear. We here propose a novel cardiovascular physiology-based weaning algorithm for MVS.

**Methods:** The proposed algorithm is based on the experience gathered at our center undergoing an Impella weaning between 2017 and 2020. Before undertaking a weaning process, patients must had been ECMO-free, afebrile, and euvolemic, with hemodynamic stability guaranteed in the absence of any inotropic support. The algorithm consists of 4 steps according to the acronym TIDE: (i) Transthoracic echocardiography under full Impella-unloading; (ii) Impella rate reduction in single 8–24 h-steps according to patients hemodynamics (blood pressure, heart rate, and ScVO_2_), including a daily echocardiographic assessment at minimal flow (P2); (iii) Dobutamine stress-echocardiography; (iv) Right heart catheterization at rest and during Exercise-testing via handgrip. We here present clinical and hemodynamic data (including LV conductance data) from paradigmatic weaning protocols of awake patients admitted to our intensive care unit with cardiogenic shock. We discuss the clinical consequences of the TIDE algorithm, leading to either a bridge-to-recovery, or to a bridge-to-permanent LV assist device (LVAD) and/or transplantation. With this protocol we were able to wean 74.2% of the investigated patients successfully. 25.8% showed a permanent weaning failure and became LVAD candidates.

**Conclusions:** The proposed novel cardiovascular physiology-based weaning algorithm is based on the characterization of the extent and sustainment of LV unloading reached during hospitalization in patients with cardiogenic shock undergoing MVS with Impella in our center. Prospective studies are needed to validate the algorithm.

## Introduction

Cardiogenic shock is a life-threatening low-cardiac-output state leading to end-organ hypoperfusion, and hypoxia (1). Despite major advances in the therapies, both medical and surgical, its in-hospital mortality remains very high (2–5). Mechanical circulatory support (MCS) for patients with advanced heart failure has flourished in the past years, supported by the availability of a multitude of devices (6). These devices have different hemodynamic peculiarities (7), depending on the insertion technique and sites of blood withdrawal and return, on the size of catheters and cannulas as well as on the need of a gas exchanger (8). Among the MCS devices, extracorporeal life support (ECLS) plays a central role in cardiogenic shock, given its reasonable cost and widespread availability in cardiology as well as in cardiothoracic surgery centers. ECLS is based on a transcutaneous performed veno-arterial cannulation, which allows to support circulation at the bedside while oxygenating blood *via* a gas exchanger (9). However, the outflow in the aorta creates an extremely high afterload, detrimental for left ventricular (LV) contractility as well as dimensions because of increased wall tension and therefore myocardial oxygen demand (8). Until a reliable strategy to unload or decompress the compromised and distended LV can be established, myocardial recovery will therefore continue to be impaired (10). In line with this, several modalities of temporarily mechanical ventricular support (MVS) for LV unloading as well as LV decompression under ECLS were investigated, such as atrial septostomy (11), the intra-aortic balloon pump (IABP) (12), the percutaneous transaortic pulsatile assist device iVAC2® (13, 14), the percutaneous continuous centrifugal pump TandemHeart® (6, 15), as well as the Impella family of devices (2.5, CP, 5.0 and 5.5, Abiomed, Danvers, Massachusetts) (16). They differ with regard to the amount of liters pumped per minute (IABP 0.5, iVAC2L, Impella 2.5 up to 5.5 l/min, TandemHeart up to 5 l/min) as well as for their mode of action (pulsatile vs. non-pulsatile). The Impella systems are transcutaneously (except from Impella 5+) deployed axial flow pumps, which directly pump blood from the LV to the ascending aorta just above the aortic valve. This approach directly unloads the LV throughout the cardiac cycle, reducing total mechanical work, and myocardial oxygen demand, while lowering wall stress and improving subendocardial coronary blood flow (8, 17). The Impella 5+ pump, which is that most frequently used in our department, is placed through an arterial cut-down (18). With flow rates of up to 5 L/min it can offer full support.

While the weaning from MCS is essentially based on the evaluation of circulatory stability of patients, weaning from MVS holds a higher complexity, being dependent on bi-ventricular function and its adaption to load. As a result of this, weaning from MVS is mostly performed in the absence of established algorithms (19–21) and strongly depends on the experience of the individual centers. Meanwhile, MVS via Impella is applied in several cardiogenic shock etiologies, such as acute myocardial infarction (AMICS, support over days) or acute fulminant myocarditis [prolonged support over weeks, PROPELLA (22)]. PROPELLA was shown to exhibit disease-modifying effects important for myocardial recovery [i.e., bridge-to-recovery) when applied in patients with acute fulminant myocarditis as a prolonged support over several weeks in awake, mobilized patients (22)]. While the weaning algorithm has been established for long-term LVAD (23), it does not exist for temporary LV mechanical support systems.

When considering the timespan of unloading, several important principles need to be followed: (i) individuality of chosen strategy, in order to avoid a one-size-fits-all approach; (ii) “Primum non-nocere,” keeping the time of mechanical unloading as short as possible, in order to prevent device-related complications; (iii) efficacy of unloading, i.e., to unload as long as needed to obtain a sustained impact on LV recovery. A weaning concept should therefore predict the likelihood of stable cardiac function when MVS is discontinued. The authors describe here their institutional experience of 141 cases with the use of the Impella device (CP or 5+) to support the LV in patients with cardiogenic shock. Fifty-two patients died during Impella support. Eighty-nine patients were tried to get weaned from the device. We here propose a practical algorithm for the weaning of cardiogenic shock patients that can be investigated prospectively in future studies. The possible outcomes of the weaning protocol are exemplified by original pressure-volume loops from invasive LV conductance measurements performed in patients admitted to the authors' center with cardiogenic shock.

## Methods and Results

The target population of this weaning concept are patients with AMICS, decompensated dilative cardiomyopathy, or acute fulminant myocarditis implanted and supported with an Impella device for several days (short time support) up to weeks [prolonged support; (PROPELLA concept) (22)]. The here proposed algorithm was therefore established in such a cohort of survivors (*n* = 89) which had been consecutively admitted between 2017 and 2020 to the intensive-care unit at the Dept. of Cardiology in Campus Virchow, Charité-Universitätsmedizin Berlin, Germany, as described in Table 1. The description of the algorithm is substantiated by clinical and hemodynamic data from paradigmatic weaning protocols of patients. The data regarding the characteristics of the patients were retrieved from an ongoing study register on the Impella weaning protocol.

**Table 1:** Population characteristics of the *n* = 89 patients weaned according to the “TIDE” algorithm.

	**Patients undergoing a TIDE-weaning (*n* = 89)**
**Age (years)**	61.6 ± 12.8
**Sex**	
-Male	59 (66.3%)
-Female	30 (33.7%)
**CVRF**	
-Arterial hypertension	44 (49.4%)
-Diabetes mellitus	(32.6%)
**Etiology**	
-Ami	46 (51.7%)
-Non-ischemic/dilative	27 (30.3%)
-Fulminant Myocarditis	16 (18%)
**EF**	
-Pre-interventional	
• 44–35%	6 (6.7%)
• 34–15%	52 (58.5%)
• <15%	31 (34.8%)
-Post-interventional	
• ≥55%	15 (16.9%)
• 55–45%	11 (12.4%)
• 44–35%	17 (19.1%)
• 34–15%	16 (18%)
• <15%	20 (22.5%)
**NT-pro BNP (ng/l)**	
-Pre-interventional	7,473.0 [2,033–17,324.5]
-Post-interventional	2,419.0 [688–10,777]
**Lactate (mg/dl)**	
-Pre-interventional	20 [12.25–39.0]
**Device**	
-Impella CP	67 (75.3%)
-Impella 5.0/5.5	22 (24.7%)
**Duration of Impella-support**	
-Short-term	4.6 ± 3.0
-Long-term (PROPELLA)	16.9 ± 6.2
**Access**	
-A. femoralis	62 (69.7%)
-A. Subclavia	27 (30.3%)
Weaning failure/LVAD-candidate	23 (25.8%)

*Parametric data are presented as mean ± SD. Non-parametric are presented as median and interquartiles. CVRF, cardiovascular risk factors; AMI, acute myocardial infarction; LVEF, left ventricular ejection fraction; LVAD, left ventricular assisted device*.

### Impella Unloading and Implications for Weaning as Assessed via LV Conductance

At the authors'institution, an exhaustive hemodynamic assessment with LV-conductance and RV-catheterization was performed in individual cases during weaning from Impella. Pulmonary capillary wedge pressure, right atrial pressure, and pulmonary pressure are determined by right heart catheterization (Swan-Ganz catheter) and pulmonary vascular resistance calculated using standard formulas. A conductance catheter (10-mm spacing, CD Leycom, Zoetermeer, The Netherlands) is used to assess continuous online measurements of LV pressure (*via* a piezoelectric membrane-based high-fidelity signal) and volume as previously described (24). This catheter is positioned along the long axis of the LV by the left femoral artery (7F). The total LV volume signal is calculated from a maximum of seven segmental volume signals, calibrated with thermodilution-derived cardiac output as well as hypertonic saline boluses as previously described and validated (25). Hemodynamic indices are obtained from steady-state PV loops during sinus rhythm and at end expiration (26).

**Figure 1:**
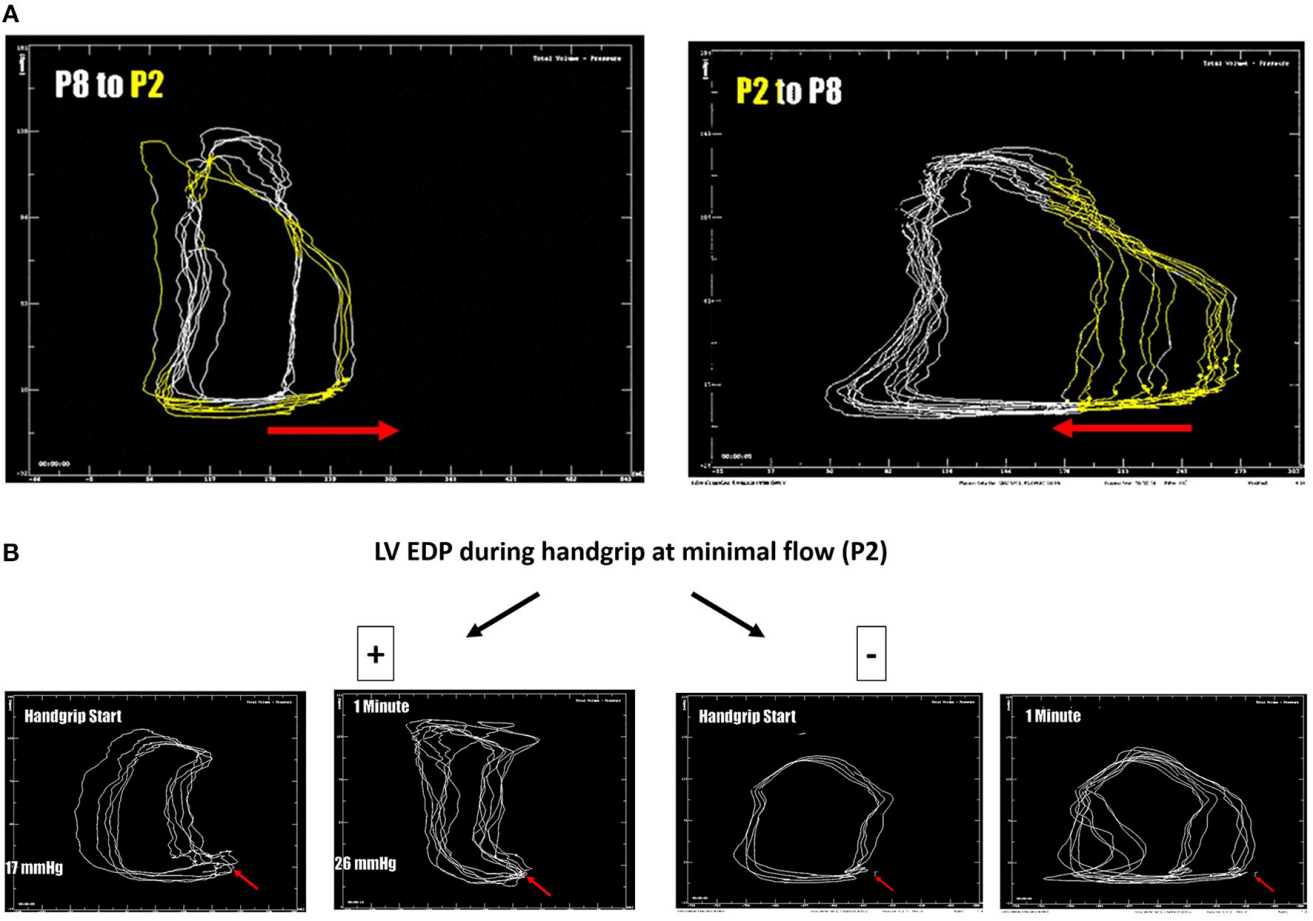
**(A)** LV pressure-volume loops (original tracings) from patients during Impella-rate reduction (P2, marked in yellow, on the left) as well as back to maximal unloading (P8, on the right). The typical shift on the xy plan is observed. **(B)** LV pressure-volume loops (original tracings) from patients during handgrip. On the right a patient who profited from unloading, showing no or minimal increase of LV end-diastolic pressure (EDP, red arrow) during handgrip; on the left a patient who had an abnormal pronounced increase of LV EDP during handgrip, as a sign of blunted contractile reserve.

**Figure 2:**
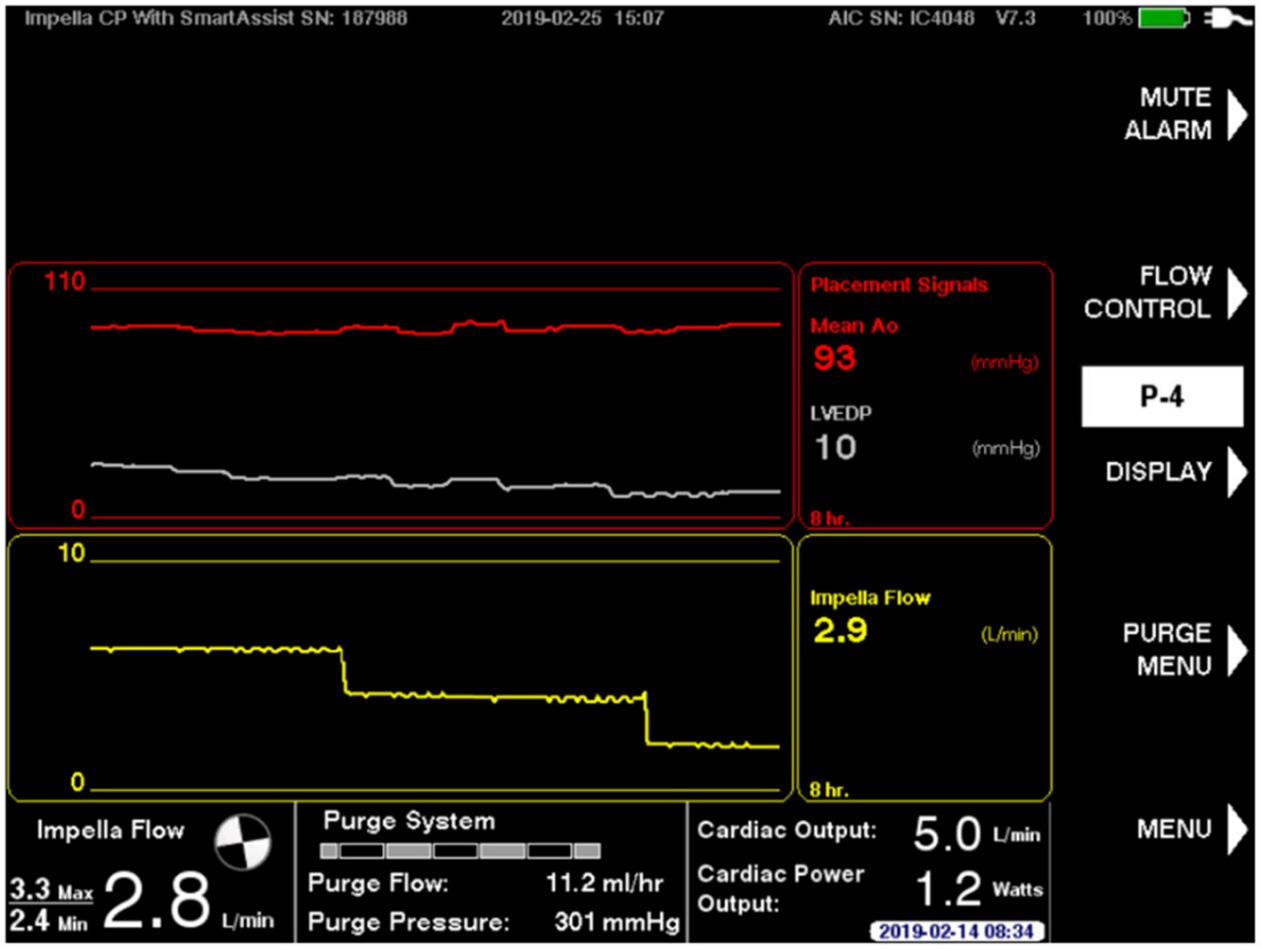
The novel Impella smart assist, displaying real-time LV end-diastolic pressure and mean arterial pressure on the Impella console.

Baseline measurements are acquired during full Impella unloading as well as during minimal Impella support (P2). To assess LV function during exercise-like conditions, steady-state PV loops are additionally acquired during handgrip at minimal Impella support over 2 min. Figure 1A (and related.gif files as online supplement) shows original tracings of Impella patients during rate change (from full unloading P8 to minimal support P2 and back) with the typical shift of the loops on the xy plane. Figure 1B (and related.gif files as online supplement) shows two patients with different study outcomes: on the right a patient who profited from unloading, showing no or minimal increase of LV end-diastolic pressure (EDP) during handgrip; on the left a patient who had an abnormal pronounced increase of LV EDP during handgrip, as a sign of blunted contractile reserve. With regard to LV EDP, a novel Impella system has been introduced on the market, called smart assist (Figure 2). This system displays real-time LVEDP, mean arterial pressure (MAP), and cardiac power output (CPO) directly on the Impella console, and will probably play an important role in the future, especially with regard to the weaning process.

### Surgical Technique for the Implantation of Impella 5.0 or 5.5

Patients are operated in the hybrid OR. Usually, the right axillary artery is dissected and a 10-mm vascular graft (Maquet Hemagard, Getinge Group, Sweden) anastomosed in an end-to side fashion. The graft is tunneled and externalized through the skin outside of the skin incision. The Impella 5.0 or 5.5 is implanted as described elsewhere under fluoroscopic guidance and echocardiographic control. The incision is closed layer by layer.

### The “TIDE” Algorithm for Weaning

**Figure 3:**
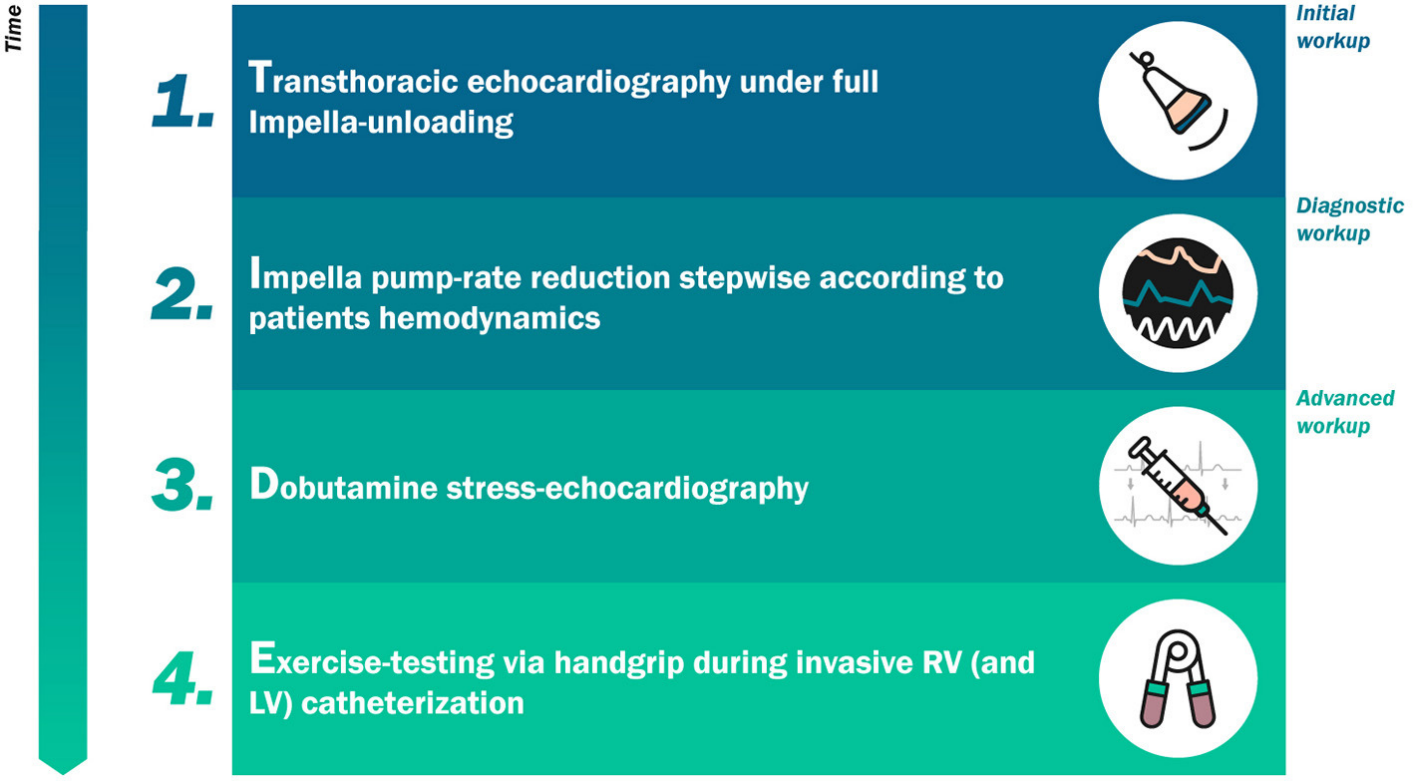
The “TIDE” algorithm for weaning from mechanical circulatory support.

Figure 3 summarizes the proposed algorithm. This algorithm consists of four chronological steps according to the acronym TIDE. The first two steps are mandatory. The latter two steps are indicated in very specific clinical scenarios. The basic principles of TIDE can be applied to either acute, intermediate or prolonged Impella support.

### Pre-requisite Before Starting With the Weaning

Before undertaking a weaning process, patients under Impella support should be afebrile and euvolemic, as reflected by standard intensive-care imaging (chest x-ray) as well as by adequate arterial PaO2. In addition, hemodynamic stability, i.e., adequate heart rate, arterial blood pressure, central venous pressure and stable rhythm, should be guaranteed in the absence of any inotropic as well as vasoactive agents, while parameters of end organ dysfunction should be fully recovered to baseline values. At the authors'institution, all echocardiographic assessments are performed with a GE Vivid S70 ultrasound system.

### Step One, Transthoracic Echocardiography Under Full Impella Unloading

Echocardiographic hemodynamic monitoring is the basic technique to assess LV functional stability for the management of patients before and during (27, 28), Impella, PROPELLA or ECMELLA weaning. It is essential to anticipate either recovery or weaning failure, a lesson learned from decision-making algorithms in ECLS (21), as well as in LVAD recipients (29). With regard to patients with ECMELLA, the weaning from ECLS is almost merely based on the evaluation of circulatory stability with ECMO flow to a minimum rate of 1.2–1.5 L/min, while the Impella support is usually discontinued in a separate weaning step.

In principle, a precise assessment of heart function is only feasible at minimal flow (P2). However, minimal flow (P2) echocardiography can be performed once the overall cardiac kinetic as well as LV-size show an improvement (LV end-diastolic diameter below 60 mm), while mitral and tricuspid regurgitation should be mild or less under full unloading. The echocardiographic evaluation consists of three steps, investigating 3 different compartments as described below:

- *Left heart function*. LV chamber dimensions should be evaluated according to the guidelines on chamber quantification (30). In addition, LV ejection fraction (LV EF) should be assessed using the biplane Simpson's method and the LV outflow tract (LVOT) velocity-time integral (VTI) using a pulsed waveform Doppler in an apical 5-chamber view. The ratio between early mitral inflow velocity and mitral annular early diastolic velocity (E/e') should be assessed.
- *Pulmonary circulation*. Tricuspid regurgitation assessment via continuous-wave Doppler with concomitant estimation of the systolic pulmonary arterial pressure (sPAP) should be performed, including estimation of mean pressure (mPAP), and pulmonary vascular resistance (PVR) by standard Doppler echocardiography formulas.
- *Right heart function*. RV dimensions and functionality should be evaluated e.g., *via* RV fractional area change (FAC), RV free wall longitudinal strain or tricuspid annular plane systolic excursion (TAPSE).

**Figure 4:**
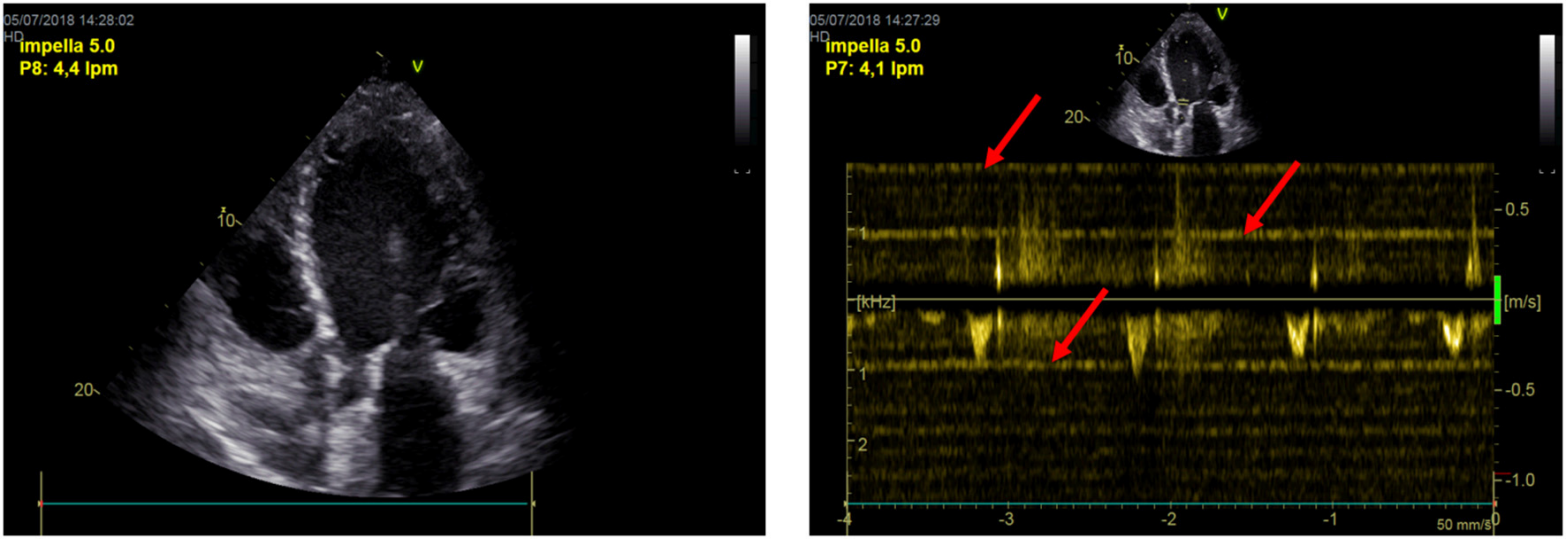
Impella's artifacts interfere in 2-dimensional echo and Doppler images of the heart.

Moving to step two of the algorithm can be considered if the patient has no end-stage cardiac disease and has partially or fully recovered from the initial cardiac dysfunction, as assessed by the abovementioned indices. Figure 4 illustrates the artifact that is introduced into the images because of the electromagnetic motor of the pump positioned in the ascending aorta. This artifact interferes in 2-dimensional Doppler images of the heart (31).

### Step Two, Impella Rate Reduction, Including Daily Minimal Flow Echocardiography

**Figure 5:**
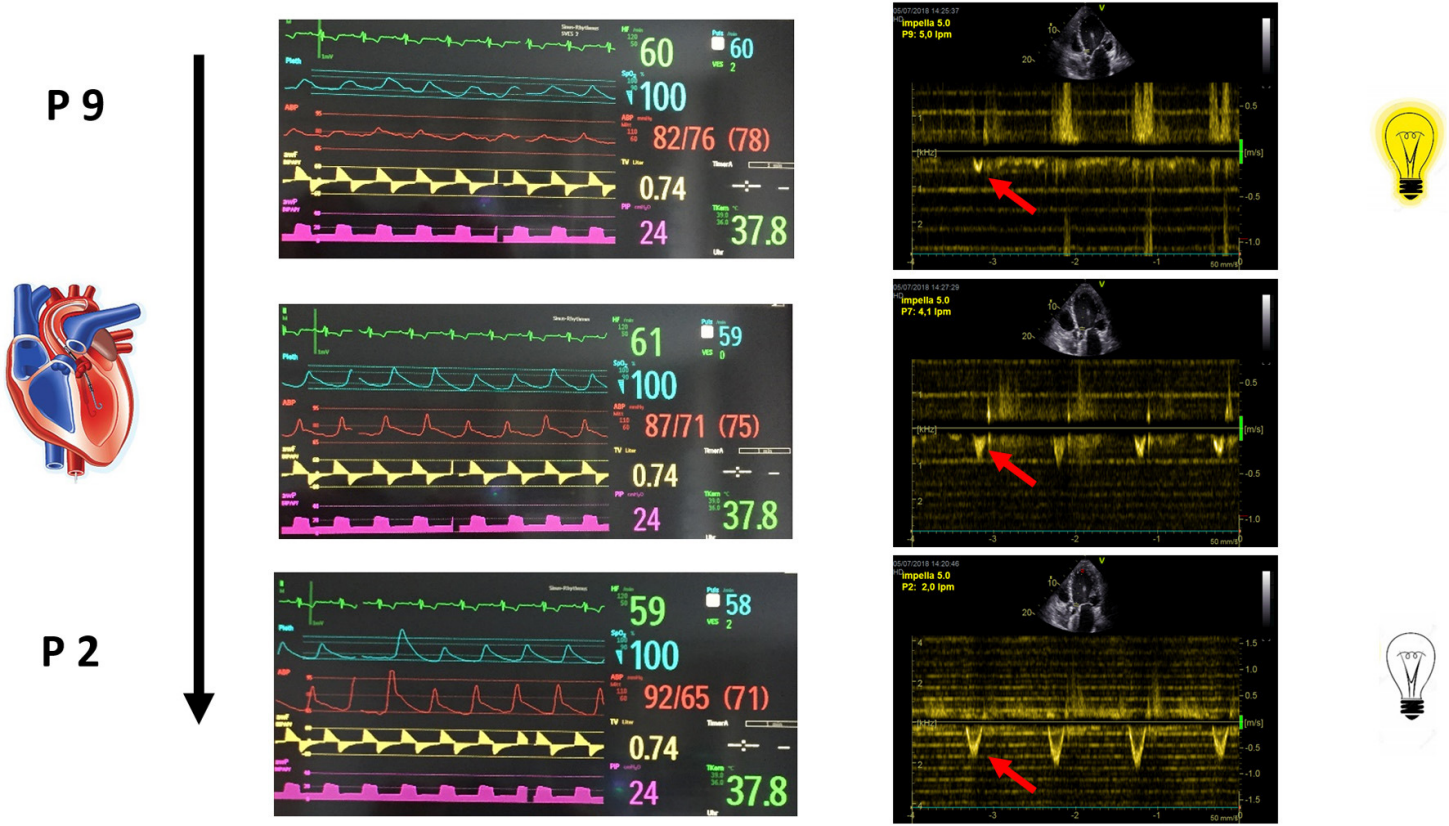
The increase in LVOT VTI with a lower level of Impella support is the Doppler echocardiographic demonstration of bi-ventricular recovery.

After step one evaluation is completed, Impella rate is decreased in single steps (e.g., P8 to P7, and so on) with the goal of achieving half of the baseline support with stable hemodynamics. At every rate, echocardiographic changes in the abovementioned three compartments as well as hemodynamic responses (blood pressure, heart rate, lactate and central venous O_2_ saturation) are monitored over the total procedure. If, at any period in the weaning protocol, RV or LV distension occurred or significant hypotension or increase in heart rate is observed, the weaning protocol is stopped and Impella support is returned back to full flow. Overall, Impella support should be reduced by single 8–24-h steps and then daily re-evaluated. During the daily, minimal flow (P2) echocardiographic examination following parameter and their acute changes will play a major role in the clinical decision-making process:

- *Left heart*. LV chamber dimensions, LV EF and E/e'. In order to discontinue the Impella support, LVEF should be above 30–35%. A LVOT VTI increase with a lower level of Impella support is the Doppler echocardiographic demonstration of bi-ventricular recovery (Figure 5).
- *Pulmonary circulation*. sPAP, mPAP, and PVR, in order to exclude an overt pulmonary hypertension.
- *Right heart*. RV dimensions, RV FAC, RV free wall LS or TAPSE, in order to evaluate long-term RV stability.

In case the three compartments show stable LV and RV function, as well as pulmonary circulation, the weaning is concluded with the discontinuation of Impella support.

In following cases, the weaning should proceed into step three of the algorithm:

- Uncertain results, i.e., discrepancy between LV EF and forward stroke volume changes during Impella results, such as an increase between 10 and 20% of forward stroke volume despite increase of LV EF above 5%, or high estimated filling pressure (E/e') or LV EDP according to the smart assist console.
- Early device-related complications, either technical such as a device defect, or clinical, such as stroke, leg ischemia, or significant hemolysis.

Step three should clarify, if a new device with a different insertion site (switch to an axillary approach) or an alternative unloading approach (e.g., iVAC2l) is needed, or the discontinuation of the Impella support is possible.

### Step Three, Dobutamine Stress-Echocardiography for Intrinsic Cardiac Reserve

**Figure 6:**
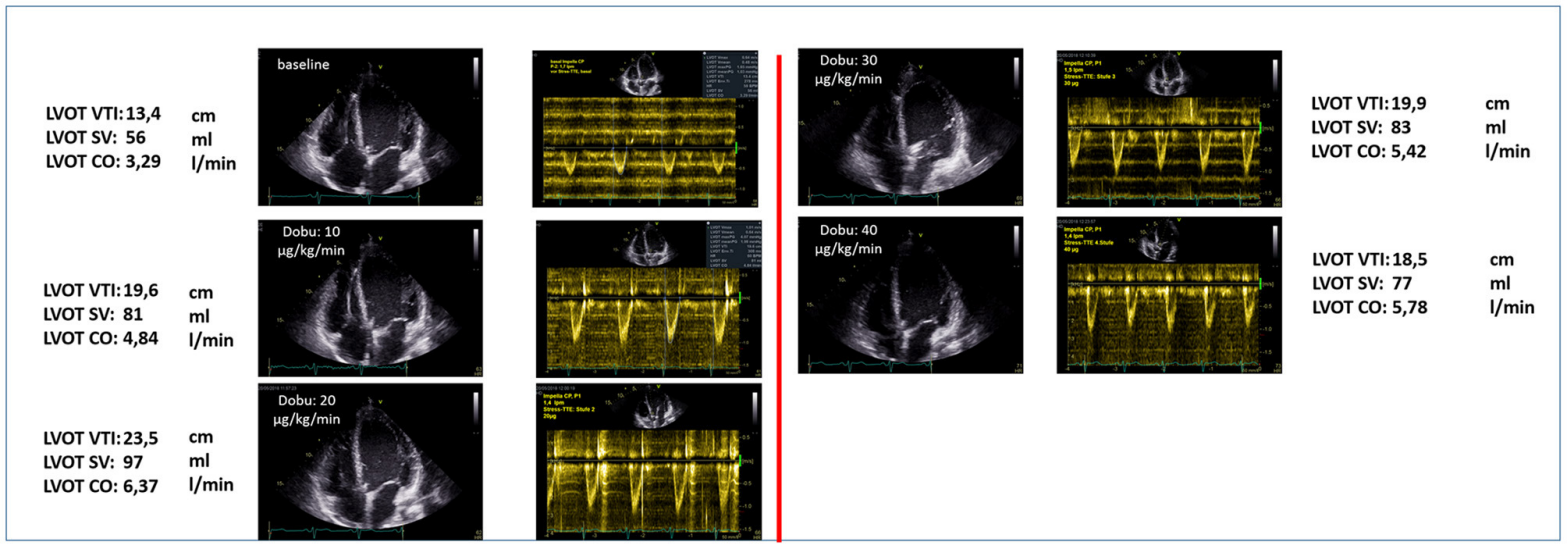
Summary of a dobutamine stress-echocardiographic examination of a patients after 4 weeks of LV unloading as a treatment for a fulminant non-viral myocarditis.

**Figure 7:**
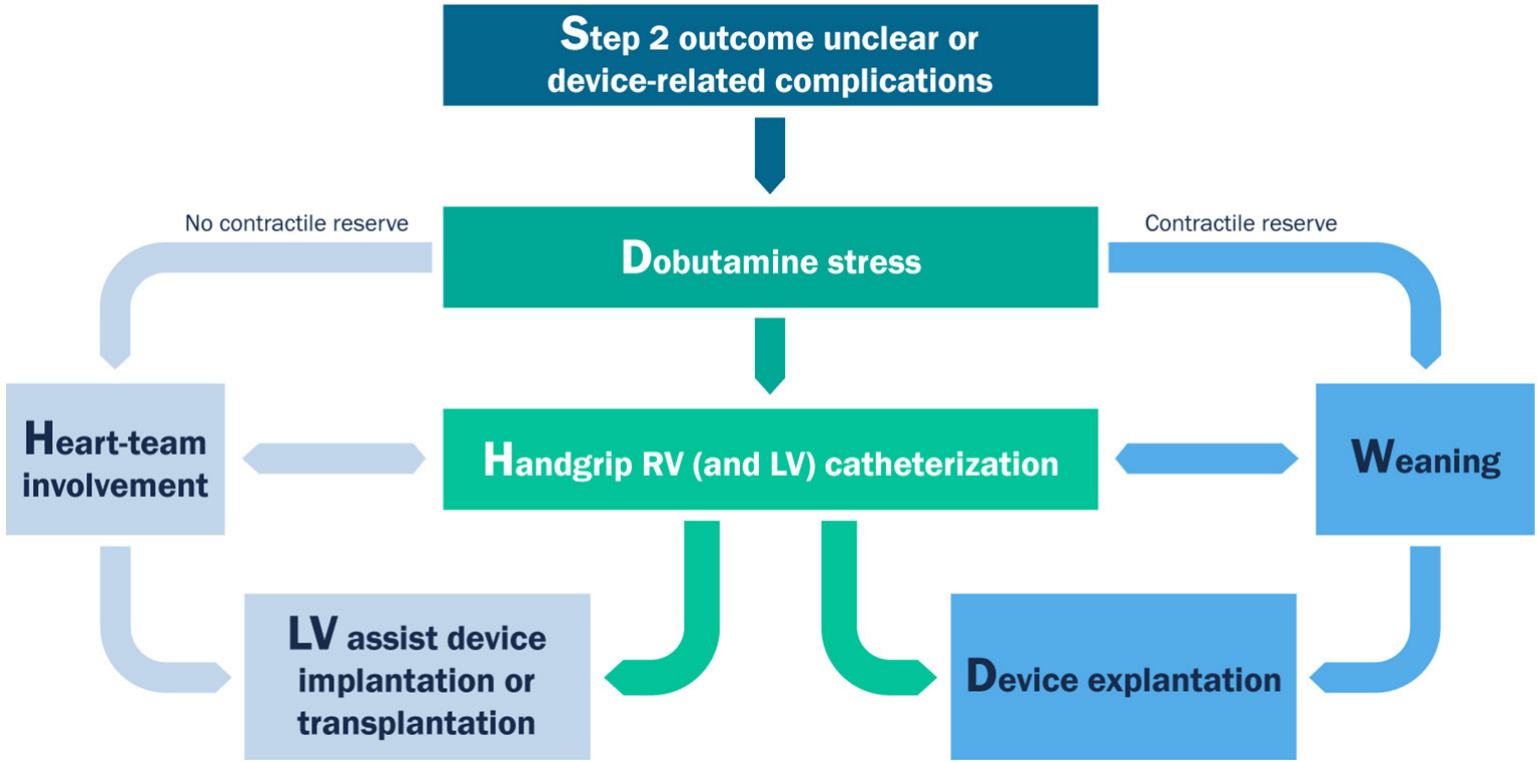
Workflow for clinical decision-making after dobutamine-stress echocardiography.

Figure 6 shows a summary of a dobutamine stress-echocardiographic examination of a patients after 4 weeks of LV unloading as a treatment for a fulminant non-viral myocarditis. For detection of inotropic response in patients during MCS, stages of 5–10 min are used, starting from 10 up to 40 μg/kg/min, in order to fully recruit the inotropic reserve in such patients under beta-blocker therapy (32). After each increment in dobutamine dose, a period of 5 min before starting the image acquisition will allow the hemodynamic response to develop. As it is not feasible to assess all possible parameters during stress, priority should be given to parameters of contractile reserve, such as changes in LV volumes, LV EF and RV EF and LVOT VTI. Global contractile reserve is often defined as an increase by >5% in LVEF while a flow reserve is defined as an increase in forward stroke volume by ≥20% (32). As the purpose of the dobutamine test is to assess contractile reserve, atropine co-administration, associated with higher rate of complications, is not indicated in MCS patients (32). In dilated non-ischaemic cardiomyopathy, patients with significant LV EF improvement during dobutamine infusion have a better survival rate, fewer hospitalizations for HF, and an increase in the LVEF during follow-up (33). The presence of inotropic contractile reserve is also associated with a decrease in the need for cardiac transplantation (34) and correlates inversely with the extent of interstitial fibrosis and scarred myocardium (35). Patients with a contractile reserve will be prepared for device removal, while patients with no reserve should be discussed within the heart-team sessions for LVAD-implantation or transplantation. In the last case, or in case of uncertain forward flow reserve, an additional invasive hemodynamic characterization (step four) should be initiated (Figure 7).

### Step Four, Exercise-Testing via Handgrip During Invasive Catheterization

In awake patients being evaluated for LVAD-implantation or transplantation, as well as blunted contractility reserve, a right-heart catheterization should be performed, including assessment of changes in cardiac index, PAP, pulmonary capillary wedge pressure (PCWP) and pulmonary vascular resistance (PVR) during handgrip compared to baseline, in order to assess cardiac and pulmonary circulation reserve. At the authors'institution, an exhaustive hemodynamic assessment including both LV-conductance and RV-catheterization is performed, as described above (Figure 1). Of note, not all the patients were awake during the beginning of weaning assessment, for this reason only the awake patients performed a handgrip during the weaning procedure.

### Outcome of TIDE-Algorithm Driven Weaning Process

A total of 141 patients with AMICS, decompensated dilative cardiomyopathy, or acute fulminant myocarditis were supported with an Impella device for several days (short time support; 4.6 + 3.0 days; Table 1) up to weeks (prolonged support; PROPELLA concept; 16.9 + 6.2 days; Table 1) (22). 89 patients underwent a weaning process. 66/89 (74.2%) passed the weaning program successfully. In about one fifth of these cases a single weaning attempt was sufficient, while the rest of patients had to undergo several attempts until the device could be safely explanted. Successful weaning was associated with a reduction of NT B-natriuretic peptides (BNP) levels and an improvement in LV EF (Table 1). 32/89 (25.8%) of patients did not pass the weaning program successfully. These patients showed a minimal change in LVEF only, despite a similar drop in NT-BNP levels compared to the successfully weaned patients (66.8 vs. 64.1%, respectively; n.s.), indicating that Impella-induced unloading was sufficient in both groups. However, even a repetition of the program did not allow a stable weaning in these cases. Thereby, most of these patients did not pass Step two of TIDE. These patients became LVAD candidates.

## Discussion

In patients with cardiogenic shock due to acute myocarditis, temporary MCS may provide bridge to durable MCS, transplant, or recovery. ECLS causes on the long-term LV dilation due to retrograde arterial flow and, therefore, increased afterload, while triggering mechanotransduction and inflammation pathways (36, 37). Left ventricular (LV) unloading by percutaneous transvalvular axial-flow devices, such as the Impella family of devices [2.5, CP, and 5+ (16)], has therefore emerged as a potential clinical breakthrough in the field. LV unloading by Impella was shown to exhibit disease-modifying effects important for myocardial recovery (i.e., bridge-to-recovery) when applied in patients with acute fulminant myocarditis as a prolonged support over several weeks in awake, mobilized patients [prolonged Impella or PROPELLA (22)]. These findings are in line with data from previous studies on the short- and median term beneficial effects at molecular, neurohormonal, cellular and structural level during LV unloading with durable devices (38). However, the duration of the “prolonged” Impella concept has not yet been standardized, i.e., the timepoint of weaning from Impella in this cohort of patients remains unclear, with most cardiac arrest centers performing weaning from MCS in the absence of clear guidelines or algorithms (19–21). The authors describe here their institutional experience with Impella-mediated LV venting in cardiogenic shock patients, while proposing a novel cardiovascular physiology-based weaning algorithm (TIDE). We discuss the clinical consequences of the TIDE algorithm, leading to either a bridge-to-recovery, or to a bridge-to-permanent LVAD and/or transplantation.

### Percutaneous Transvalvular Axial-Flow Devices in Cardiogenic Shock

Percutaneous unloading using a transaortic LV assist device in cardiogenic shock has become increasingly popular and has therefore been widely adopted in the last years. Most of data in literature are derived from studies focusing on acute myocardial infarction complicated by cardiogenic shock (AMICS). However, a strong clinical evidence of the use of such devices based on randomized multicentric trials is still missing. A recently published analysis of outcomes for 15,259 US patients with acute myocardial infarction complicated by cardiogenic shock supported with the Impella device, showed a wide variation in outcomes across included centers. Survival was higher when Impella was used as first support strategy, when combined with invasive hemodynamic monitoring, and at centers with higher Impella implantation volume (>7 Impella cases per year). In hospitals in the bottom quintile of Impella implantation volume, mortality was 70%, but only 24% in the highest quintile (39). Of note, the systematic use of shock protocols positively impacted on survival in AMICS, probably because of a more appropriate selection of the timing of device implantation. In a small, single-center, non-randomized study Impella CP initiation prior to PCI was associated with higher survival rates at discharge and up to 1 year in AMICS patients presenting with high risk for in-hospital mortality (40). Furthermore, unloading the left ventricle before reperfusion in STEMI was feasible as well as safe (41). The utilization of Impella in cardiogenic shock after myocardial infarction was also associated with less complications compared to ECLS (42). Finally, the strategy of percutaneous LV unloading using a transaortic LV assist device in combination with VA-ECLS improved outcome in an all-comers cohort compared to what predicted by established risk scores, such as SAVE or SAPS-II (43).

### Evidence on Early-Stage LV Unloading in Acute Myocarditis

Acute fulminant myocarditis is characterized by a transient contractility impairment, which accounts not only for the tissue damage but rather for a myocardial stunning similar to the one after reperfusion in AMI. While stunning is reversible, it can take several days to weeks to resolve (44). It is the result of an oxidative stress on the ground of microvascular ischemia and an unfavorable remodeling due to inflammation as well as to increased mechanical wall stress (36). Based on the consistent finding of good long-term survival outcomes for acute fulminant myocarditis, an aggressive hemodynamic support in the early stages of the disease seems to consistently impact on long-term survival outcomes, being therefore crucial (16, 45). MCS not only offers a bridge to recovery but also could hasten the process of recovery. Several mechanisms are involved in promoting myocardial recovery in the setting of mechanical circulatory support. The most important is related to the increase of perfusion pressure through increasing coronary flow which seems to play a central role in VA-ECMO therapy (46). In line with that, LV unloading via a percutaneous device such as Impella, could reduce the wall stress not only via a reduction of microvascular resistance but also reducing the mechanical work while minimizing the myocardial oxygen demand (44). Although epidemiological data demonstrate an increase in the incidence of cardiogenic shock secondary to myocarditis during the last decade, the mortality remained the same, possible due to the increased utilization of mechanical circulatory support (45).

### Before Starting With the Weaning Trial

The determination of the ideal conditions to start the weaning trial is the key point for the outcome and has been relatively underestimated by the studies so far. The existing data are mainly based on ECLS studies, where the mean duration of support was ranging from 3 up to 8 days (20, 47). Of importance, the main factor which allows the beginning of a weaning trial is the reversibility of the etiology that led to cardiogenic shock (48). Therefore, therapeutic interventions such as reperfusion, specific myocarditis therapy or electrical stabilization should have been performed to improve the course of cardiogenic shock (49). Furthermore, the patient should have recovered from the hemodynamic conditions which lead to MCS, i.e., high volume overload as well as high dose of inotropic agents (49), having a baseline mean arterial pressure (MAP) of >60 mmHg in the absence or at low doses of vasoactive agents and a pulsatile arterial waveform maintained for at least 24 h (49–52). According to the Extracorporeal Life Support Organization (ELSO) guidelines, recovery of hepatic function is essential before to perform any attempt to wean patients from ECLS (52). However, it does not seem necessary to wait for the recovery of kidney function (53).

### Predicting Weaning Success

As the use of MCS in cardiogenic shock increases, the identification of reliable indicators of successful MCS weaning constitutes an issue in patient selection and treatment planning. The existing data derive from observational studies with patients who underwent ECLS support and durable LVAD (47). Diverse clinical, echocardiographic, invasive, and non-invasive hemodynamic parameters as well as biomarkers have been evaluated. Small studies have assessed the ability of pulse pressure (5) and functional parameters of sublingual microcirculation [such vessel density, (54)] to predict successful weaning. Among the biomarkers only lactate and lactate clearance are reported to be associated with outcome (55). Furthermore, invasive markers (off pump values) such as cardiac index >2.4l/min/m2, pulmonary capillary wedge pressure <18 mmHg (56) and cardiac power output CP > 0.6 W, CPI>0.4 W/m^2^ (56) were showed to correlate well with myocardial recovery. Echocardiography plays a central role in the weaning process. Parameters reflecting the systolic function of left and right ventricle such as aortic velocity time integral, LVEF, mitral annulus peak systolic velocity, 3D-RVEF as well as RV free wall strain own a high predictive value. Additionally, LV and RV dilation were found as independent negative predictive factors. The use of the aforementioned indicators to assess the success of weaning in patients with Impella is relatively arbitrary and reveals the need of more robust evidence in the context of randomized trials.

### Weaning Strategies: The Role of Echocardiography and Invasive Catheterization

The knowledge gained in the field so far is derived from two echocardiography based ECLS weaning studies, one based on transthoracic (50) and one on transoesophageal (57) examination. In the first one (50), as soon as the patient was considered hemodynamically and respiratory stable, ECLS support was gradually reduced to 66% initially and then to 33% of initial support for 10–15 min and finally to a minimum of 1–1.5 l/min. In case of blood pressure drop the weaning trial was discontinued and ECLS flow returned to a full support. Criteria to remove the ECLS were functional recovery and good toleration of weaning trial, meaning a LVEF >20% and an aortic VTI >10 cm.

The second weaning study (57) consisted of four steps, in line with the protocol described in the current work. Initial baseline RV and LV function and volumes were measured under full ECLS support. In the second step the ECLS support was gradually decreased in single steps of 0.5 l/min with the goal of achieving the half of the initial flow with maintenance of adequate hemodynamics. At every flow level, RV and LV function, dimensions and hemodynamic responses were monitored over 5–10 min to allow estimation of ventricular function and volume status. If, at any period in the weaning protocol, RV or LV distension occurred or significant hypotension or increase in heart rate was observed, the weaning protocol was stopped. and ECLS support was returned to full flow. The third step included volume challenge with albumin 5% meanwhile the ECLS support was reduced at a minimum of 1.2–1.5 l/min. In last step LV an RV function was evaluated under inodilators (dobutamine or milrinone over 4–6 h) in order to evaluate the RV function before LVAD implantation. In case of recovery of RV and LV function ECLS could be removed. It is worth noting, that the weaning protocol should be adjusted depending on the type of mechanical support, as ECLS leads to overloading of the left ventricle and unloading of the right ventricle, while a percutaneous transvalvular approach such as with the Impella unloads mainly the left ventricle. ECLS weaning protocols should therefore include a careful assessment of the function of the right ventricle and aim at preserving hemodynamic stability under minimal mechanical support, while weaning protocols from an Impella support should focus on evaluation of left ventricular function and contractile reserve, as described in Figure 7.

While several studies in literature have described the effect of most common MCS and MVS on LV and RV hemodynamics by pressure-volume analysis (8, 17, 58), exhaustive conductance catheter investigation of biventricular function do not play a major in the weaning process from MCS/MVS. This is certainly due to the low availability of the system in real-world centers, as well as for the invasive approach needed for such an investigation. In line with this, the pressure-volume data from individual patients reported in the current manuscript are to be understood as physiology-based insight in the unloading process and its potential outcomes.

### Limitations

The proposed algorithm was developed at a single institution and needs further clinical evidence. The validation of the TIDE algorithm and the analysis of the predictive factors that may identify the candidate of LVAD implantation are the subject of an ongoing study and were not the purpose of the current manuscript. It cannot be used for right ventricular weaning strategies. It had not been investigated in other LV unloading devices. Outcome of patients and weaning success highly depends on the experience of the single center with the device being utilized. As a large university hospital, we cannot exclude a selection bias, and we therefore here underline the need for multi-center studies.

### Conclusion

The proposed novel cardiovascular physiology-based weaning algorithm is based on the characterization of the extent and sustainment of LV unloading reached during hospitalization in patients with acute fulminant myocarditis and cardiogenic shock undergoing MCS with a percutaneous transvalvular axial flow device. However, its validity in the context of several types of MCS, as well as of different pathogenesis of cardiogenic shock, needs further investigation.

## Data Availability Statement

The original contributions presented in the study are included in the article/supplementary material, further inquiries can be directed to the corresponding author.

## Ethics Statement

Written informed consent was obtained from the individual(s) for the publication of any potentially identifiable images or data included in this article.

## Author Contributions

AA, FS, CT, GS, and HP designed the clinical protocols. AA, AF, FS, CT, and GS conducted the investigations. AA, AF, CT, and FS prepared the manuscript. All authors revised the manuscript.

## Conflict of Interest

CT, FS, and GS received honoraria for talks from Abiomed. The remaining authors declare that the research was conducted in the absence of any commercial or financial relationships that could be construed as a potential conflict of interest.
